# A Magnesium Phosphate-Based Platform Alleviates Bone–Fat Imbalance for the Repair of Age-Related Osteoporotic Bone Defects

**DOI:** 10.3390/biomedicines14061302

**Published:** 2026-06-08

**Authors:** Xiping Zhang, Yue Luo, Ye Liu, Wenda Liu, Jian Zheng, Changtian Gong

**Affiliations:** 1 Department of Orthopedics, Renmin Hospital of Wuhan University, Wuhan 430060, China; zhangxp@whu.edu.cn (X.Z.); rm001873@whu.edu.cn (Y.L.); 2018305230061@whu.edu.cn (W.L.); 2 The Central Hospital of Yichang, No. 183 Yiling Avenue, Wujia Gang District, Yichang 443003, China; 3 Center of Regenerative Medicine, Renmin Hospital of Wuhan University, Wuhan 430060, China; 2024103020053@whu.edu.cn

**Keywords:** magnesium phosphate, bone–fat imbalance, osteoporosis, bone defects repair

## Abstract

**Background/Objectives**: Osteoporosis poses significant obstacles as it causes an imbalance between osteoblasts and adipocytes, which results in the disruption of bone homeostasis. Although various magnesium-based scaffolds have been deployed for the treatment of osteoporotic bone defects, whether this can be achieved by alleviating bone–fat imbalance still requires further elucidation. **Methods**: We designed magnesium phosphate-based platforms (GMPCs), based on magnesium photopolymerized methacrylated gelatin (GelMA) and phosphate (K-struvite, MPC), and used them to deliver magnesium ions (Mg^2+^) for alleviating bone–fat imbalance locally. **Results**: The in vivo results demonstrated that the GMPCs not only improved osteogenic behavior at the implanted site, but also reduced the proportion of adipose tissues in a femoral defect model in 18-month-old SD rats. Moreover, by promoting the differentiation of bone marrow mesenchymal stem cells (BMSCs) into osteoblasts in a concentration-dependent manner, GMPCs significantly reduced adipogenic differentiation in vitro. Also, 5GMPC demonstrated the best comprehensive biologic properties compared to other platforms. **Conclusions**: GMPCs have great potential in the treatment of age-related osteoporosis via the effective delivery of Mg^2+^.

## 1. Introduction

Osteoporosis (OP) is a metabolic bone disease characterized by decreased bone mass and microstructure destruction [1,2]. The pathogenesis of OP is mainly related to the dysfunction of osteoblasts and osteoclasts, leading to an imbalance between bone resorption and bone formation, and this impairs the quality of life [3]. Thus, bone defect repair within OP poses challenges to achieving initial stability with hindered bone integration and regeneration ability.

As the most common complication resulting from OP, current treatments for osteoporotic bone defects (OPDs) mainly fall into two categories: pharmacologic therapy and surgery [4,5]. Pharmacologic therapy mainly focuses on general treatment to reduce the risk of fractures, especially for postmenopausal women and elderly people [6]. For example, according to the Women’s Health Initiative, estrogen has potent anti-resorptive properties in bone and reduces vertebral and non-vertebral fracture risk. And surgery is commonly performed to relieve pain after complications have occurred. At this stage, various types of bone cement are used to reconstruct biomechanical stability [7,8]. However, there is no method that can combine the advantages of the above two methods simultaneously.

Recently, beyond conventional approaches, magnesium has been considered a critical cue in the pathogenesis of OP [9]. Two critical regulators, parathyroid hormone (PTH) and vitamin D, are affected by magnesium deficiency and can impair natural bone structures, indicating that magnesium is involved in the occurrence and development of OP [10,11]. As reported, PTH regulates the formation of osteoclasts by stimulating the expression of receptor activator of nuclear factor κB (RANK) and receptor activator of nuclear factor κB ligand (RANKL). Additionally, magnesium may influence vitamin D3-mediated bone remodeling, which typically coordinates the activation balance between osteoblasts and osteoclasts [12,13,14]. But, when osteoporosis occurs, an increased amount of bone marrow fat tissue is observed, and the way in which magnesium participates in this has not yet been fully clarified.

In this study, we observed the variation in adipose tissue within the bone marrow of aged SD rats. And based on this, we created a femoral defect model to mimic the complications of OP. Next, our designed magnesium phosphate-based platforms (GMPCs) were implanted to achieve locally sustained release of Mg^2+^, which has already been proven to be effective in a skull defect model. Furthermore, as a complement to the in vivo experiments, we evaluated the osteogenic and adipogenic differentiation behaviors in a cellular OP model in vitro. In summary, this study provides more evidence for how magnesium can alleviate age-related OP by regulating bone–fat imbalance.

## 2. Materials and Methods

### 2.1. In Vivo Experiments

#### 2.1.1. Preparation of Animal Models and GMPC Composites

All animal experiments were approved by the Animal Welfare and Ethics Committee of Renmin Hospital of Wuhan University (WDRM20210121). All rats were kept in a pathogen-free environment with a standard diet and ad libitum access to food and water. Rats were accommodated in group housing under standard conditions of 20–24 °C and 40–60% humidity, following a 12 h/12 h light/dark cycle. For bone–fat imbalance assessment, 18-month-old SD female rats were selected and set as the OLD group, and 12-week-old SD female rats were set as the YOUNG group.

Based on previous research, the same formula and group set were used again in this study [2]. Briefly, MPC powders were mixed with basic GelMA (5%, *w*/*v*) on the basis of the weight-to-volume ratio (g mL^−1^, 2.5%, 5%, and 7.5%). Then, UV curing was performed (405 nm, 20 s) to get cross-linked composites, named 2.5GMPC, 5GMPC, and 7.5GMPC, respectively. Then, basic GelMA without MPC addition was set as the control, as appropriate.

Next, according to the same parameters above, twenty 18-month-old SD female rats were prepared and divided into five groups of four rats each: Sham, GelMA, 2.5GMPC, 5GMPC, and 7.5GMPC groups. The SD rats were anesthetized using sodium pentobarbital (30 mg/kg), and their operative areas were disinfected afterwards. The skin was incised along the medial side of the knee joint to reveal the femur. An area of bone defect with a diameter of 4 mm and a depth of 6 mm was created. All composites were then implanted after sterilization with ethylene oxide. After the surgery had been completed, the incision was sutured in a layer-by-layer manner. After the operation, marks were made on the ears of each rat for subsequent observation and management.

#### 2.1.2. Histological Examination

To further evaluate the osteogenic and adipogenic effects, the femur samples from YOUNG, OLD, and material-implanted groups were all subjected to HE staining, Masson staining, Goldner staining, and immunohistochemical staining of FABP4 and OCN, respectively. At the pre-designated time point (8 weeks), the animals were sacrificed. Afterwards, according to standard procedures, the samples were fixed with 4% paraformaldehyde and decalcified with EDTA to observe the changes in bone tissue morphology. The specific steps and parameters used can be referred to in the published paper [2].

As stated above, pathological analysis of the major organs was conducted to determine the in vivo biological safety. Specifically, tissues from the hearts, lungs, spleen, liver, and kidneys were obtained and stained with the HE staining kit to observe the major structure variation.

#### 2.1.3. Micro-CT

At designated times (8 weeks), the harvested femoral specimens were subsequently immersed in 4% neutral buffered formalin for fixation. High-resolution micro-computed tomography (micro-CT, skyscan1276, Bruker, Kontich, Belgium) was used. The imaging acquisition parameters were configured as follows: pixel size of 18 μm, tube potential of 50 kv, tube current of 500 μA, per-projection exposure duration of 100 ms, and an angular increment of 0.9° per eight projections. The region of interest (ROI) was inside the femoral defect. Reconstruction and presentation were performed via image software to get the quantitative assessment data. The following t indices were obtained: Bone volume fraction (BV/TV), bone mineral density (BMD), trabecular thickness (Tb.Th), and trabecular number (Tb.N).

### 2.2. In Vitro Experiments

#### 2.2.1. Isolation and Identification of Rat Bone Marrow Mesenchymal Stem Cells (BMSCs)

Female SD rats (4–6 weeks old) were euthanized via an intraperitoneal overdose injection (tribromoethane, 0.3 mL/100 g). Each rat was positioned in dorsal recumbency under aseptic conditions, and hindlimbs were both dissected to get the tibiae. The epiphyseal ends of the tibiae were incised, and the marrow cavity was flushed with L-DMEM supplemented with 10% fetal bovine serum (FBS) and 1% penicillin-streptomycin repeatedly. The cell suspension was filtered through a 200 μm cell strainer and centrifuged at 1500 rpm for 5 min. After discarding the supernatant, the cell pellet was resuspended, seeded into culture flasks, and maintained under standard conditions at 37 °C with 5% CO_2_.

Firstly, a medium change was performed at 48 h post-seeding to remove non-adherent cells with the adherent population retained. Then, cell culture was continued until 80–90% confluence was observed, followed by dissociation with 0.25% trypsin-EDTA with further passaging. After passaging three to four times, a morphologically homogeneous population of fibroblast-like mesenchymal stem cells (MSCs) was obtained.

Next, for immunophenotypic identification, third-passage cells (P3) were subjected to flow cytometry using fluorescence-conjugated antibodies against positive (e.g., CD90, CD44, CD105) and negative (e.g., CD34, CD45) surface markers. After incubation, cells were acquired on a flow cytometer. The isolated cell population was defined as BMSCs only when the expression rates of positive markers exceeded 95% and those of negative markers were below 2%. These validated BMSCs were subsequently used for other experiments.

#### 2.2.2. Construction of Cellular OP Model

After exposure to dexamethasone (1 μmol/L) for 6 h, bone marrow mesenchymal stem cells (BMSCs) exhibited phenotypic characteristics consistent with an in vitro cellular OP model [15]. Untreated BMSCs served as the control group. In all subsequent cellular experiments in this study (excluding the initial model validation experiments), the dexamethasone-induced osteoporotic BMSC model was uniformly employed to ensure experimental consistency.

#### 2.2.3. Preparation of Extracts from GMPCs and Osteogenesis/Adipogenesis Culture Solution

After 72 h of co-culture with GMPCs at a ratio of 200 mg/mL, extracts were collected after filtration with a 200-mesh filter, and stored at 4 °C for further use. The osteogenic and adipogenic induction media were all purchased from Procell (PD-008 and PD-013), and the above two liquids were mixed in a 1:1 volume ratio to prepare the osteogenesis culture solution and adipogenesis culture solution, respectively.

#### 2.2.4. Immunofluorescent Staining

Based on the cellular OP model, a total of 5 × 10^4^ dexamethasone-induced BMSCs were seeded with osteogenesis/adipogenesis culture solution in a confocal culture dish. After 7 or 14 days, BMSCs were rinsed with phosphate-buffered saline (PBS) twice and fixed in 4% paraformaldehyde (PFA) for 20 min. Subsequently, 0.3% Triton X-100 was used to permeabilize cells for 10 min at room temperature. To block non-specific binding, a blocking buffer consisting of 5% bovine serum albumin (BSA) was applied at 37 °C for 30 min. After that, the blocking buffer was removed, and the primary antibody (OCN, 1:200; COL-1, 1:200, FABP4, 1:300, 100 μL/dish) was added, followed by incubation overnight at 4 °C. Afterwards, BMSCs were retrieved and a fluorophore-conjugated secondary antibody (Alexa Fluor^®^ 594 Goat anti-rat IgG, 100 μL/dish) was applied and incubated for 1 h at room temperature in the dark. Nuclear staining was performed by adding DAPI solution (G1012, Servicebio, Wuhan, China) for 5 min, and cytoskeleton staining was stained with phalloidine (G1248, Servicebio). Finally, image acquisition was performed using a laser scanning confocal microscope (MDI8, Leica, Wetzlar, Germany).

#### 2.2.5. BODIPY Staining

As stated above, a total of 5 × 10^4^/well dexamethasone-induced BMSCs were seeded on 6-well plates, and cultured with adipogenesis culture solution for 14 days. Afterwards, BMSCs were washed twice with PBS and cultured with PFA for 10 min. Then, according to manuals, working solution was added into the 6-well plates (1 mL/well), followed by 10 min incubation at room temperature in the dark. Eventually, image acquisition was performed using a laser scanning confocal microscope (MDI8, Leica, Germany).

#### 2.2.6. Oil Red O Staining

The preparation process for the cell samples was the same as that for BODIPY staining. After 14 days, the medium was removed, and the BMSCs were stained with Oil red O staining solution (C0157S, Beyotime, Shanghai, China) for 30 min, followed by two PBS washes. The images were captured using an inverted fluorescence microscope (Olympus, IX71, Tokyo, Japan) and relative positive areas (%) were calculated using ImageJ (1.8.0).

### 2.3. Statistical Analysis

Randomization: Animals were randomly assigned to groups using a random number table. Blinding: All micro-CT, histological, and biomechanical analyses were performed in a blinded manner. The sample size of n = 4 per group was determined based on effect sizes from previous similar studies in bone defect models. The statistical power for the primary endpoints (bone volume fraction, osteoblast number) reached *p* < 0.05.

All data were analyzed using GraphPad Prism 10 and presented as mean ± standard deviation (SD). Differences between two groups were assessed using the *t*-test and multiple comparisons were performed using one-way ANOVA. Significance levels are indicated as * *p* < 0.05, ** *p* < 0.01, and *** *p* < 0.001.

## 3. Results and Discussion

The core pathology of OP is the imbalance of bone homeostasis between bone formation and bone resorption [16,17,18]. Existing evidence has suggested that multiple signaling pathways are involved, mainly including the classical RANKL/RANK/OPG signaling pathway and the Wnt/β-catenin signaling pathway [4,19]. Consequently, designing therapeutic strategies targeting the above mechanisms to achieve a balance between osteoblasts and osteoclasts has became a central part of managing OP. However, in age-related OP, the loss of bone mass is accompanied with an increased proportion of adipose tissue, implying that bone–fat imbalance is also involved in the occurrence of age-related OP.

**Figure 1:**
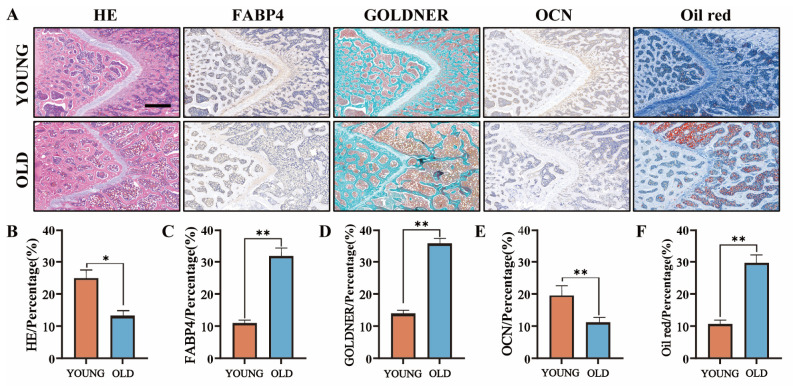
Histological staining of YOUNG and OLD groups. (**A**) HE staining, IHC staining of FABP4, Goldner staining, IHC staining of OCN, and Oil red staining. (**B**–**F**) Quantitative analysis of HE staining, IHC staining of FABP4, Goldner staining, IHC staining of OCN, and Oil red staining. The scale is 100 μm in Figure 1A. Significance levels are indicated as * *p* < 0.05, ** *p* < 0.01.

As presented in Figure 1A, representative histological staining images of the femur were selected from the YOUNG and OLD groups. When compared to the YOUNG group, the HE staining and Goldner staining both showed that the trabecular structure in the OLD group was disordered and the bone volume was reduced, and IHC staining of OCN and FABP4 indicated that the expression level of adipogenesis and osteogenesis displayed an opposite trend, which was the same as previously reported. Moreover, Oil red staining showed that there were more lipid droplets in the bone marrow of the OLD group and that fat deposition was significantly increased. The above results were all consistent with the related quantitative analysis shown in Figure 1B–F.

Over decades, magnesium delivery systems have received extensive attention in the treatment of age-related bone loss [20,21,22]. Lu et al. have developed porous microsphere scaffolds to enhance OP-related bone regeneration, which could continuously release hydrogen, magnesium ions (Mg^2+^), and dimethyl fumarate (DMF) to inhibit pyroptosis by activating nuclear factor erythroid related factor 2 (NRF2) and reshape the inflammatory microenvironment within osteoblasts and osteoclasts [23]. Zhao et al. created a microfluidic GelMA-BP-Mg microsphere with bone-targeting abilities to activate osteoblasts while restraining osteoclasts, and finally achieved the reconstruction of OP-affected bone tissues [24]. However, whether Mg^2+^ could promote OP-related bone regeneration by reducing fat tissues still remains to be elucidated. In our previous study, based on GelMA, through the gradient addition of K-struvite, magnesium phosphate-based platforms (GMPCs) were developed, indicating the satisfactory delivery capacity of Mg^2+^ to promote bone regeneration in cranial defects of SD rats. Thus, in this work, we continuously used GMPCs.

**Figure 2:**
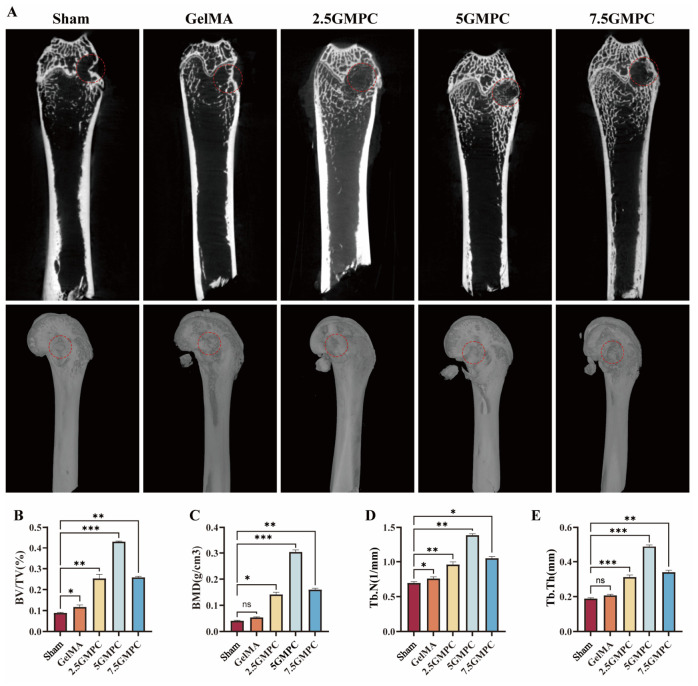
3D reconstruction of the femoral defect model in 18-month-old rats after being implanted for 8 weeks. (**A**) Representative X-ray and 3D reconstruction images. (**B**–**E**) Quantitative analysis of bone volume fraction (BV/TV), bone mineral density (BMD), bone trabecular number (Tb.N), and bone trabecular thickness (Tb.Th). Significance levels are indicated as * *p* < 0.05, ** *p* < 0.01, and *** *p* < 0.001.

After implantation for 8 weeks, we conducted micro-CT as the assessment method for newly formed bone tissues. Representative X-ray and 3D reconstruction images are illustrated in Figure 2A, and, as expected, the 5GMPC group possessed the highest amount of new bone tissue inside the range of interest (ROI), which represented the same trend in BV/TV, BMD, and Tb.N to Tb.Th (Figure 2B–E). Previously, biodegradation properties for all GMPCs have been proven, and the concentration of Mg^2+^ released in a sustained manner from 5GMPC has been found to be between 5 and 15 mmol/L [2], reaching the optimal concentration. We speculated that this is the main reason for the differences among OP-related bone regeneration found in this work.

**Figure 3:**
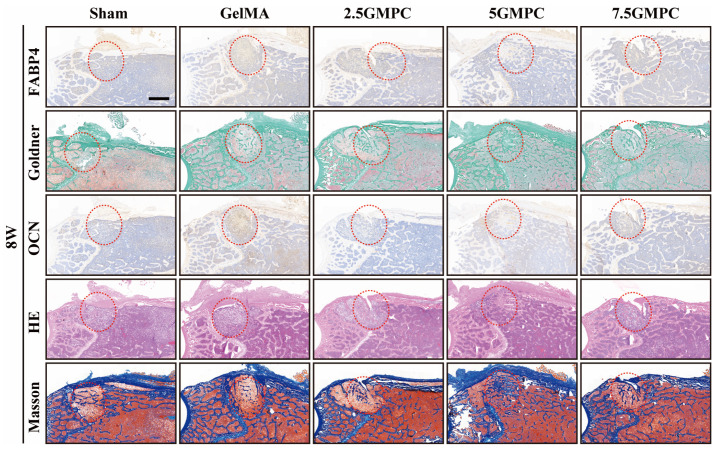
Histological staining of the femoral defect 8 weeks postsurgery, including IHC staining of FABP4, Goldner staining, IHC staining of OCN, HE staining, and Masson staining. Scale of 100 μm.

As supplementary information, the histological analysis results of the femoral bone defect 8 weeks postsurgery are also displayed. As shown in Figure 3, significantly different trends were observed among all groups under different staining methods: 1. The IHC staining of FABP4 indicated the lowest level of adipogenesis in the 5GMPC group, and there was almost no obvious infiltration of adipose tissue inside the ROI, suggesting that an appropriate concentration of Mg^2+^ can effectively inhibit adipogenic differentiation. 2. Goldner staining showed that the new bone area in the 5GMPC group was the largest out of all the groups, indicating the strongest osteogenic activity. The above trends were verified by HE and Masson staining. 3. The IHC staining of OCN showed an opposite trend to the results of IHC staining of FABP4, suggesting that the appropriate concentration of Mg^2+^ not only promotes osteogenesis but also inhibits adipogenesis in vivo. Li et al. have reported that magnesium-containing nanozymes (Mg-ZIF) can effectively inhibit the differentiation of BMSCs into adipocytes via bone marrow cavity injection [25]. In this study, we have demonstrated that similar effects can also be achieved through the in situ fixation of GMPCs, and the consistency of the effective concentration of Mg^2+^ is maintained.

**Figure 4:**
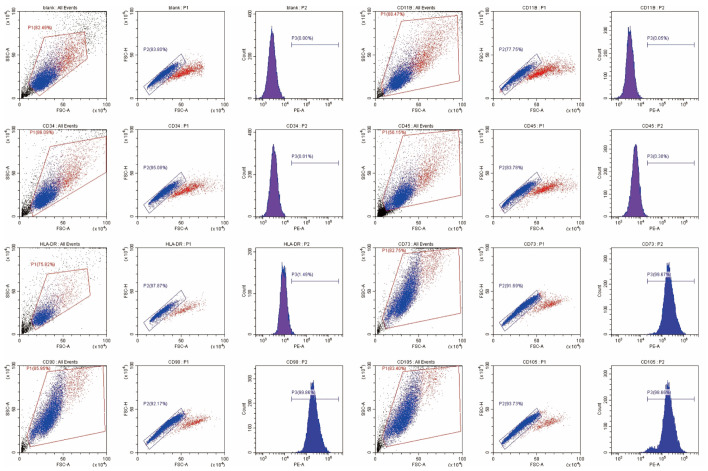
Flow cytometry results for rat bone marrow mesenchymal stem cell identification. The identification indicators from top to bottom and from left to right are as follows: negative control, CD11b, CD34, CD45, HLA-DR, CD73, CD90, and CD105.

The local milieu surrounding age-related OP is marked by a two-pronged dysregulation, as follows: diminished osteogenic capacity of bone marrow-derived mesenchymal stem cells (BMSCs) alongside an aberrant shift toward adipocytic lineage specification, which means BMSCs are the critical cues responsible for maintaining bone–fat balance [26,27,28,29]. Thus, BMSCs were extracted and identified to establish an in vitro cellular OP model in this work. The flow cytometry results for the identification of BMSCs are shown in Figure 4. The data indicate that the extracted BMSCs have good purity, containing a negative control group, TEST: 0%, and BMSC groups, CD34: 0.01%; CD45: 0.3%; CD11b: 0.05%; HLA-DR: 1.49%. CD73: 99.67%; CD90: 99.86%; and CD105: 98.66%. These results meet the internationally recognized standards for mesenchymal stem cell identification and are suitable for subsequent experiments in vitro.

**Figure 5:**
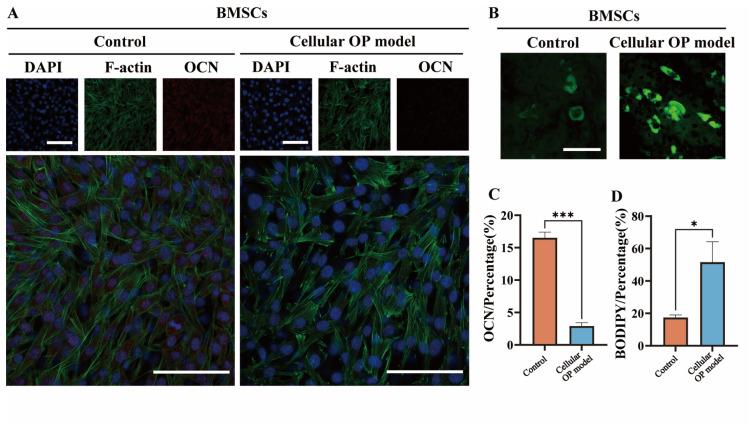
Verification of cellular OP model in vitro. (**A**,**C**) Immunofluorescence staining of OCN and quantitative analysis in the control group and the cellular OP model group. (**B**,**D**) BODIPY staining and quantitative analysis in the control group and the cellular OP model group. The scale is 100 μm. Significance levels are indicated as * *p* < 0.05 and *** *p* < 0.001.

In this work, through referring to published papers, we have established a cellular OP model for the in vitro assessment [15]. Also, immunofluorescence staining of OCN and BODIPY staining were carried out to verify the efficiency of the model. As depicted in Figure 5A,B, the cellular OP model displayed enhanced fluorescence intensity of OCN and the accumulation of lipid droplets when compared to the control group. The above results have been confirmed by quantitative analysis, as shown in Figure 5C,D. Traditionally, in the establishment of OP models in vitro, the aging of BMSCs affects its osteogenic and adipogenic differentiation capabilities. Thus, there are significant differences in the response to external ion stimulation. In this work, we used dexamethasone to induce the same batch of BMSCs, which can ensure the consistency of osteogenic and adipogenic differentiation behaviors after the intervention with the extracts.

**Figure 6:**
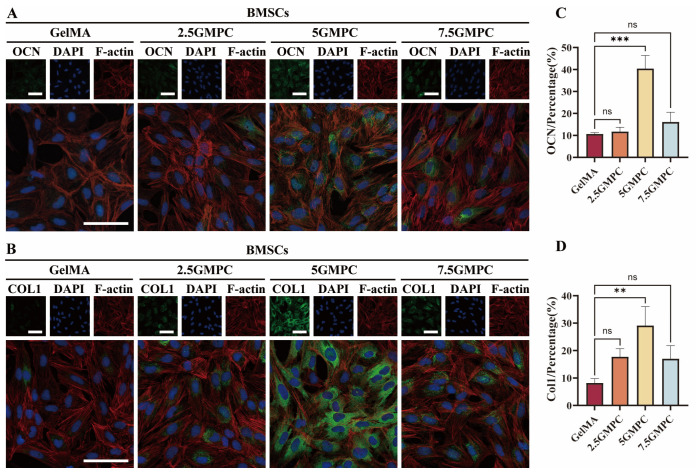
Verification of osteogenesis ability in vitro. (**A**,**C**) Immunofluorescence staining of OCN and quantitative analysis based on cellular OP model. (**B**,**D**) Immunofluorescence staining of COL-1 and quantitative analysis based on cellular OP model. The scale is 100 μm. Significance levels are indicated as ** *p* < 0.01 and *** *p* < 0.001, ns means no significant difference.

Osteocalcin (OCN) and collagen type I (COL-1) are two critical biomarkers for evaluating osteogenic differentiation: OCN specifically indicates late mineralization, while COL-1 mainly participates in the early formation of the bone matrix [30,31,32,33]. Figure 6A presents the immunofluorescence staining results of OCN. It can be seen that in the MPC, the green fluorescence signal of OCN gradually strengthened with increased content, especially in the 5GMPC group, showing the strongest osteogenic differentiation tendency with proper magnesium ion delivery. The quantitative analysis result in Figure 6C illustrates that the expression level of OCN in the GelMA group and the 2.5GMPC group is similar, with no significant difference, while the OCN-positive rate in the 5GMPC group significantly increased. Moreover, the green fluorescence signal of COL-1 exhibited a similar trend to that of OCN in Figure 6B, also indicating that the ability to synthesize collagen type I has significantly improved, while the signal intensity in the 7.5GMPC group decreased. Figure 6D further confirms that the positive rate of COL1 in the GelMA group is the lowest, slightly increased in the 2.5GMPC group, and significantly enhanced in the 5GMPC group. In summary, magnesium ions can promote differentiation towards osteogenesis and enhance the expression of OCN and COL1, and the effect is optimal in the 5GMPC group, which exerts the best osteogenic promoting effect in regulating extracellular matrix deposition.

**Figure 7:**
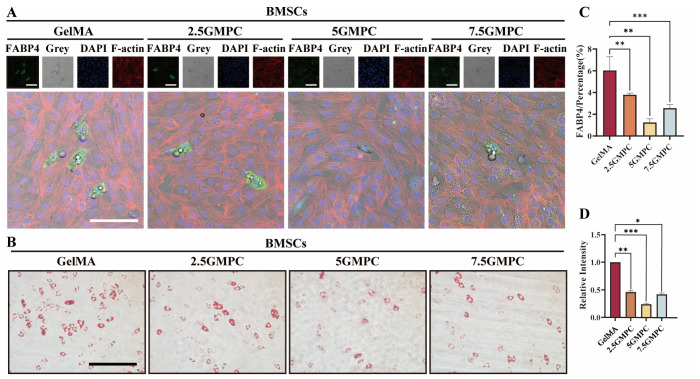
Verification of adipogenesis ability in vitro. (**A**,**C**) Immunofluorescence staining of FABP4 and quantitative analysis based on cellular OP model. (**B**,**D**) Oil red O staining and quantitative analysis based on cellular OP model. Scale of 200 μm. Significance levels are indicated as * *p* < 0.05, ** *p* < 0.01, and *** *p* < 0.001.

As another direction of cell differentiation among age-related OP, FABP4 serves as a marker for adipogenesis [34,35]. As shown in Figure 7A, there was strong positive expression in the GelMA group, indicating significant adipogenic differentiation without magnesium ions. When the MPC content increased, the FABP4 fluorescence signal weakened, suggesting that magnesium ions can inhibit adipogenesis. The merged images clearly show that FABP4-positive cells were mainly located in the cytoplasm. Also, the Oil red O staining images in Figure 7B present the results of lipid droplets within different groups. In the GelMA group, a large number of red lipid droplets can be observed, but the magnesium ions significantly reduced the number of red lipid droplets within the cells and weakened the staining intensity, suggesting that the lipidization process was inhibited. The quantitative analysis result in Figure 7C,D confirms the above results.

Previous studies have mainly focused on the regulatory role of magnesium ions in the osteoblast–osteoclast axis, which is differentiated from mesenchymal cell lineages and monocyte cell lineages [36]. In this work, we have demonstrated that by regulating the differentiation of BMSCs, reducing adipogenic differentiation can also be an effective strategy for treating age-related OP.

**Figure 8:**
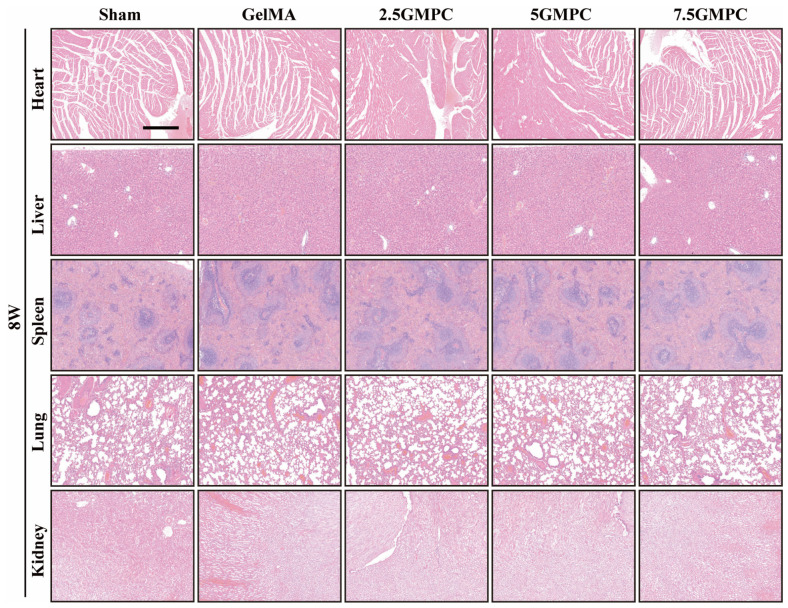
HE staining of the heart, liver, spleen, lung, and kidney from all GMPC groups at 8 weeks postoperation. Scale of 100 μm.

After 8 weeks, the rats were sacrificed and the heart, lungs, liver, spleen, and kidneys from all GMPC groups were obtained for HE staining. As shown in Figure 8, all major organs maintained normal structural organization, as follows: 1. the cardiac muscle fibers were arranged regularly; 2. the liver presented a typical hepatic lobule structure; 3. the white and red pulp of the spleen were clearly visible; 4. the alveolar structure of the lungs was intact without obvious inflammation or exudation; 5. and the renal glomeruli and renal tubules had normal structures. No obvious pathological changes were observed, indicating that all GMPCs had good biocompatibility in vivo and did not cause significant systemic toxic reactions or organ damage.

In our previous studies, we have already discussed the concentration effect of the Mg^2+^ released from GMPCs. Regarding the concentration-dependent efficacy of Mg^2+^, a biphasic dose response has been well documented in multiple studies. For example, Paiva et al. reported that 5–10 mM Mg^2+^ promotes osteogenesis, while 25 mM inhibits it and 50 mM induces cytotoxicity [37]. Zhang et al. also demonstrated that Mg^2+^ enhances osteogenic differentiation in a concentration-dependent manner, with excessive concentrations reducing cell viability [38]. Importantly, Yuan et al. systematically reviewed the concentration- and stage-dependent behavior of Mg^2+^ in bone regeneration and explicitly proposed that 2–10 mM Mg^2+^ in vitro represents the optimized concentration window [39]. Collectively, the observation that the 5GMPC outperforms the 7.5GMPC in our study is consistent with this well-established biphasic effect.

Recent advances in magnesium-based biomaterials for osteoporotic bone regeneration have provided an instructive context for our findings. While numerous studies have demonstrated that Mg^2+^ promotes osteogenesis via the Wnt/β-catenin pathway and inhibits osteoclastogenesis via the OPG/RANKL axis, thereby correcting the classical osteoclast–osteoblast imbalance, a growing body of evidence reveals a more nuanced picture concerning bone–fat imbalance [40]. Intriguingly, Amara et al. reported that high-purity magnesium implants, despite enhancing peri-implant bone formation, unexpectedly induced marrow adipogenesis accompanied by low-grade persistent inflammation, suggesting that Mg-based materials may inadvertently exacerbate the bone–fat imbalance in the bone marrow compartment [41]. This observation resonates with the core theme of our study, namely, that pathological bone–fat imbalance (skewed BMSC differentiation toward adipogenesis at the expense of osteogenesis) is a critical driver of osteoporosis. Mechanistically, PPARγ is the master switch controlling BMSC fate, and its activation suppresses osteogenesis while promoting adipogenesis. Moreover, DEPTOR was recently identified as a molecular gatekeeper of bone–fat balance by inhibiting RUNX2 and facilitating PPARγ-mediated transcription [26]. Collectively, these findings from the Mg biomaterial field underscore two important points. First, even materials that globally favor bone formation may locally perturb the bone–fat equilibrium, highlighting the necessity of evaluating not only the osteoclast–osteoblast axis but also the adipogenic side effects when designing osteoporotic bone grafts. Second, the signaling pathways identified in our study—particularly the upstream regulators of BMSC lineage commitment—may overlap with those modulated by Mg^2+^ (e.g., Wnt/β-catenin, PPARγ, and mechanotransduction pathways). Future comparative studies are therefore warranted to determine whether the bone–fat imbalance induced by magnesium implants shares common upstream mechanisms with the osteoporotic bone–fat imbalance characterized here, and whether combinatorial strategies (e.g., alloying with anti-adipogenic agents or surface functionalization) can reconcile osteopromotion with suppression of marrow adiposity.

### Limitations of This Work

While the phenotypic and histological data presented in this study provide robust evidence for the observed effects, we acknowledge a limitation regarding the mechanistic depth of the current work. Specifically, we did not identify the upstream signaling pathways responsible for transducing the initial stimuli to downstream effectors. The current dataset, though informative at the phenotypic and tissue architecture levels, does not allow us to pinpoint which upstream regulators (e.g., receptor tyrosine kinases, G-protein-coupled receptors, or intracellular signaling nodes) are involved. Consequently, the complete molecular cascade underlying our findings remains incompletely understood. To address this knowledge gap and to further validate and expand upon the present findings, we plan to conduct systematic mechanistic experiments in future studies, including quantitative PCR (qPCR), Western blotting, and RNA sequencing (RNA-seq). These approaches will help identify the upstream signaling pathways and their cross-talk, thereby providing a more comprehensive view of the regulatory network. Notwithstanding this limitation, we believe that the current phenotypic and histological evidence, together with the transparent discussion of this caveat, provides a solid foundation for the conclusions drawn in this manuscript.

## 4. Conclusions

We used previously synthesized GMPC scaffolds to deliver magnesium ions, thereby re-establishing the microenvironment and bone–fat balance within age-related OP. And by using BMSCs stimulated by dexamethasone, we demonstrated that the 5GMPC could increase the levels of osteogenesis biomarkers (OCN, COL1) and inhibit the levels of adipogenesis markers (FABP4) in a dose-dependent manner. In the femoral defect model of aged rats, the 5GMPC significantly promoted bone regeneration, which was confirmed by micro-CT and different histological stains, and also showed excellent systemic biocompatibility in vivo. In summary, these findings revealed that the appropriate delivery mode of Mg^2+^ can simultaneously correct the pathological bias between bone–fat imbalance, thereby providing a new strategy for treating age-related OP.

## Data Availability

The original contributions presented in this study are included in the article. Further inquiries can be directed to the corresponding author.

## References

[1] Ball A.N., Donahue S.W., Wojda S.J., McIlwraith C.W., Kawcak C.E., Ehrhart N., Goodrich L.R. (2018). The challenges of promoting osteogenesis in segmental bone defects and osteoporosis. J. Orthop. Res..

[2] Zhang X., Gong C., Wang X., Wei Z., Guo W. (2024). A Bioactive Gelatin-Methacrylate Incorporating Magnesium Phosphate Cement for Bone Regeneration. Biomedicines.

[3] Zhou X.Y., Jiang J.H., Dang J.B., Wang Y.L., Hu R.B., Shen C., Zhao T.H., Sun D.H., Wang G.B., Zhang M. (2024). Intelligent Supramolecular Modification for Implants: Endogenous Regulation of Bone Defect Repair in Osteoporosis. Adv. Mater..

[4] Etani Y., Ebina K., Hirao M., Kitaguchi K., Kashii M., Ishimoto T., Nakano T., Okamura G., Miyama A., Takami K. (2020). Combined effect of teriparatide and an anti-RANKL monoclonal antibody on bone defect regeneration in mice with glucocorticoid-induced osteoporosis. Bone.

[5] Li C.T., Ma C., Zhuo X.L., Li L., Li B., Li S.J., Lu W.W. (2022). Focal osteoporosis defect is associated with vertebral compression fracture prevalence in a bone mineral density-independent manner. Jor Spine.

[6] Nozaka K., Miyakoshi N., Mita M., Shimada Y. (2023). The successful treatment of a Gustilo-Anderson type IIIc distal leg injury with a large bone defect in elderly patient with severe osteoporosis: A case report. J. Med. Case Rep..

[7] Oh W.T., Yang Y.S., Xie J., Ma H., Kim J.M., Park K.H., Oh D.S., Park-Min K.H., Greenblatt M.B., Gao G.P. (2023). WNT-modulating gene silencers as a gene therapy for osteoporosis, bone fracture, and critical-sized bone defects. Mol. Ther..

[8] Tan D., Li Q.L., Chen Z.Z., Zhang H.B., Rao P.C., Li J.X., Tao Q.K., Xiao J.G., Song J.L. (2025). YTHDC1 Modulates the Osteogenic Capacity of hPDLSCs via Wnt/β-Catenin Signalling Pathway for the Treatment of Bone Defects in Osteoporosis Rats. Cell Prolif..

[9] You Y.H., Wei S.Y., Gao Z.L., Wang L.Y., Cheng Q., Chang M.Z., Ma Q.L., Wang L.L., Hu X.Z., Liu X.Y. (2026). Engineered Poly(ethylene glycol)-Alendronate-Magnesium Hydrogels Potentiate Site-Specific Immunomodulation for the Healing of Osteoporosis Fractures. Adv. Funct. Mater..

[10] Sandomierski M., Stachowicz W., Patalas A., Grochalski K., Grabon W., Voelkel A. (2023). Characterization of Magnesium and Zinc Forms of Sodalite Coatings on Ti6Al4V ELI for Potential Application in the Release of Drugs for Osteoporosis. Materials.

[11] Zittermann A. (2013). Magnesium deficit—Overlooked cause of low vitamin D status?. BMC Med..

[12] Shi D.L., Li X.Y., Men N., Ren B.W., Ma Y.F., Wang L.S., Zhang Z.B., He K.G., Du X.Z., Wang J.L. (2026). Serum magnesium is associated with osteoporosis risk in postmenopausal women: A retrospective study and risk-prediction model. Front. Med..

[13] Weng Z.Z., Ye J., Cai C.X., Liu Z.K., Liu Y.Y., Xu Y.Y., Yuan J.H., Zhang W., Liu L.B., Jiang J.K. (2024). Inflammatory microenvironment regulation and osteogenesis promotion by bone-targeting calcium and magnesium repletion nanoplatform for osteoporosis therapy. J. Nanobiotechnol..

[14] Sun P., Wang M., Yin G.Y. (2020). Endogenous parathyroid hormone (PTH) signals through osteoblasts via RANKL during fracture healing to affect osteoclasts. Biochem. Biophys. Res. Commun..

[15] Yan C., Zhang P., Qin Q., Jiang K., Luo Y., Xiang C., He J., Chen L., Jiang D., Cui W. (2024). 3D-printed bone regeneration scaffolds modulate bone metabolic homeostasis through vascularization for osteoporotic bone defects. Biomaterials.

[16] Unwanatham N., Disthabanchong S., Ponthongmak W., Prechaporn W., Assanatham M., Nimitphong H., Sritara C., Thakkinstian A. (2026). Accelerated bone loss increases osteoporosis and fracture risk in moderate to severe chronic kidney disease. Ren. Fail..

[17] Qiu X.L., Wu L.J., Jiang F.X., Shan H.J., Sheng L., Tian B., Wang H., Cui H., Tao L.D., Wu C.Y. (2026). Engineered probiotic-derived indole-3-propionic acid inhibits ubiquitination via AHR signaling to treat postmenopausal osteoporosis. Gut Microbes.

[18] Qiu S.W., Ji P.H., Wang Y.M., Duan Q.Y., Yu J.D., Luo M.Q., Wu P., Huo M.F., Shi J.L. (2026). Osteoimmune-regulative metal-organic framework nanomedicine for effective osteoporosis therapeutics. Biomaterials.

[19] Cai C.H., Zhang Z.Q., Zhao X., Yang C., Huang X.M., Tang C., Qiu H., Yang S.Z., Zhang Y., Hu X. (2026). A novel METTL3 inhibitor nimbolide ameliorates osteoporosis via orchestrating osteoclastogenesis in an m6A-dependent manner. Phytomedicine.

[20] Yang K., Zhang B.Z., Zhang Y.Y., Wang X.J., Yuan X. (2026). SHED-derived exosomes ameliorate age-related osteoporosis by activating mitophagy in senescent bone marrow mesenchymal stem cells. Nanomedicine.

[21] Shao X.Y., Zhang P., Fan Z.D., Lin J.Q., Chen X., Liu N., Gong W., He Y., Zhou Y.N., Shi T.S. (2026). Atrophic Skeletal Muscle-Derived Extracellular Vesicles Transfer miR-125a-5p to Inhibit Bone Formation in Osteoporosis during Aging. Adv. Sci..

[22] Li P., Liang Z.W., Zeng X.Y., Lei R.B., Guo S., Zhang Z., Zhang G.W., Li J.X., Qin A.H., Qu M. (2026). Age-related GSS promoter methylation in BMSCs drives osteoporosis and the reversal by targeted GSH delivery. Bioact. Mater..

[23] Lu S.Y., Cao J., Song Z.R., Gong F., Yang P., Ge J., He Y.F., Han Z.H., Hou G.H., Zhang Z.M. (2025). Pyroptosis-responsive microspheres modulate the inflammatory microenvironment to retard osteoporosis in female mice. Nat. Commun..

[24] Zhao Z.Y., Li G., Ruan H.T., Chen K.Y., Cai Z.W., Lu G.H., Li R.M., Deng L.F., Cai M., Cui W.G. (2021). Capturing Magnesium Ions Microfluidic Hydrogel Microspheres for Promoting Cancellous Bone Regeneration. ACS Nano.

[25] Li J., Chen Y., Zha D., Wu C., Li X., Yang L., Cao H., Cai S., Cai Y. (2024). Mg-ZIF nanozyme regulates the switch between osteogenic and lipogenic differentiation in BMSCs via lipid metabolism. Lipids Health Dis..

[26] Xiong Z., Song Y., Wu J.F., Lu W., Xiong J.J., Zhu M.L., Wang L.H., Chen Y., Xu L., Li X.N. (2025). Bidirectional regulation factor of bone marrow mesenchymal stromal cells differentiation: A focus on bone-fat balance in osteoporosis. Stem Cell Res. Ther..

[27] Chen M.M., Liang H., Wu M., Ge H.Y., Ma Y., Shen Y., Lu S.Y., Shen C.L., Zhang H.X., Wang Z.G. (2024). Fgf9 regulates bone marrow mesenchymal stem cell fate and bone-fat balance in osteoporosis by PI3K/AKT/Hippo and MEK/ERK signaling. Int. J. Biol. Sci..

[28] Li L., Wang B., Li Y.W., Li L., Dai Y.L., Lv G.H., Wu P.F., Li P.Z. (2020). Celastrol regulates bone marrow mesenchymal stem cell fate and bone-fat balance in osteoporosis and skeletal aging by inducing PGC-1α signaling. Aging.

[29] Yu B., Huo L.H., Liu Y.S., Deng P., Szymanski J., Li J., Luo X.H., Hong C., Lin J.D., Wang C.Y. (2018). PGC-1α Controls Skeletal Stem Cell Fate and Bone-Fat Balance in Osteoporosis and Skeletal Aging by Inducing TAZ. Cell Stem Cell.

[30] Wu T.L., Yang G.Y., Yuan Z., Xu R., Yang X.W., Jiawei H., Liao X.P., Zhang B. (2026). Bone-Targeting Gallium-Gallic Acid Metal-Organic Framework (GGMA) for Dual Anti-Inflammation and Osteo-Regeneration Therapy of Osteoporosis. ACS Appl. Mater. Interfaces.

[31] Li H.T., Pan H.Y., Feng M.S. (2026). Enhancing osteoporosis treatment: Emerging roles of engineered exosomes in bone regeneration and repair. J. Transl. Med..

[32] Leung S., Li X.H., Huang X.Q., Xu R.G., Wu H.K., Deng J.L., Deng F.L., Guo S.Z., Liu Y. (2026). Dual-controlled release of PTH(1-34) via microsphere-hydrogel scaffold promotes early bone regeneration in osteoporosis. Colloid Interface Sci. Commun..

[33] Meng L., Zhao P.P., Jiang Y.C., You J.W., Xu Z.Y., Yu K., Boccaccini A.R., Ma J.Q., Zheng K. (2024). Extracellular and intracellular effects of bioactive glass nanoparticles on osteogenic differentiation of bone marrow mesenchymal stem cells and bone regeneration in zebrafish osteoporosis model. Acta Biomater..

[34] Xie Q., Du X.F., Liang J.H., Shen Y.N., Ling Y.F., Huang Z.J., Ke Z.K., Li T., Song B., Wu T.L. (2025). FABP4 inhibition suppresses bone resorption and protects against postmenopausal osteoporosis in ovariectomized mice. Nat. Commun..

[35] Liu H.R., Xiong Y.Q., Zhu X.F., Gao H., Yin S.J., Wang J.F., Chen G.M., Wang C.P., Xiang L., Wang P.P. (2017). Icariin improves osteoporosis, inhibits the expression of PPAR, C/EBP, FABP4 mRNA, N1ICD and jagged1 proteins, and increases Notch2 mRNA in ovariectomized rats. Exp. Ther. Med..

[36] Dai B.Y., Li X., Xu J.K., Zhu Y.W., Huang L., Tong W.X., Yao H., Chow D.H.K., Qin L. (2021). Synergistic effects of magnesium ions and simvastatin on attenuation of high-fat diet-induced bone loss. Bioact. Mater..

[37] Paiva S.S., Ferreira A., Pakenham E., Kaur K., Cavanagh B., O’Brien F.J., Murphy C.M. (2025). Magnesium Ion-Mediated Regulation of Osteogenesis and Osteoclastogenesis in 2D Culture and 3D Collagen/Nano-Hydroxyapatite Scaffolds for Enhanced Bone Repair. J. Funct. Biomater..

[38] Zhang Z., Gong N., Wang Y., Xu L., Zhao S., Liu Y., Tan F. (2024). Impact of Strontium, Magnesium, and Zinc Ions on the In Vitro Osteogenesis of Maxillary Sinus Membrane Stem Cells. Biol. Trace Elem. Res..

[39] Yuan Z., Wan Z., Gao C., Wang Y., Huang J., Cai Q. (2022). Controlled magnesium ion delivery system for in situ bone tissue engineering. J. Control. Release.

[40] Zhu Y.C., Jia G.Z., Yang Y.F., Weng J., Liu S., Zhang M.W., Zhang G., Qin H.T., Chen Y.X., Yang Q. (2023). Biomimetic Porous Magnesium Alloy Scaffolds Promote the Repair of Osteoporotic Bone Defects in Rats through Activating the Wnt/β-Catenin Signaling Pathway. ACS Biomater. Sci. Eng..

[41] Ben Amara H., Martinez D.C., Iskhakova K., Emanuelsson L., Norlindh B., Loo A.J., Wieland D.C.F., Zeller-Plumhoff B., Willumeit-Römer R., Plocinski T. (2025). Multifaceted bone response to immunomodulatory magnesium implants: Osteopromotion at the interface and adipogenesis in the bone marrow. Biomaterials.

